# From mechanics to mobilization: a biomechanically-informed rehabilitation framework for cesarean-related scars

**DOI:** 10.1186/s12905-026-04702-w

**Published:** 2026-07-20

**Authors:** Ying Zhou, Jun Yao Huang, Wei Shi

**Affiliations:** 1 Department of Obstetrics and Gynecology, West China Second Hospital, Sichuan University, No.20, Section 3, Renmin South Road, Chengdu, 610041 China; 2 Key Laboratory of Birth Defects and Related Diseases of Women and Children, Sichuan University, Ministry of Education, Chengdu, China

**Keywords:** Cesarean section, Scar, Cesarean-related scars, Biomechanics, Rehabilitation, Mechanotherapy, Physical therapy

## Abstract

**Background:**

Cesarean delivery often results in both abdominal wall scars and uterine scars. However, current rehabilitation strategies for cesarean-related scars lack standardization and a biomechanically informed framework that integrates superficial (abdominal wall) and deep (uterine niche) scar management.

**Objective:**

To synthesize the pathomechanical basis of cesarean-related scarring and to propose a stage-specific, biomechanically informed rehabilitation framework, while explicitly acknowledging the evidence gap between superficial and deep scar management.

**Methods:**

A systematic literature search was conducted in PubMed, Web of Science, and Scopus from inception to August 2025, supplemented by hand-searching of reference lists. A total of 2,138 records were identified; after duplicate removal and screening, 345 full-text reports were assessed, of which 101 studies met the inclusion criteria. Studies on cesarean scar healing, adhesion biomechanics, mechanotransduction, assessment tools, and physiotherapeutic interventions were synthesized. Evidence was classified by level (I–IV) based on study design, adapted from the Oxford Centre for Evidence-Based Medicine hierarchy. A clear distinction was maintained between interventions applicable to superficial (abdominal wall) scars and those hypothetically extended to deep (uterine niche) defects.

**Results:**

Aberrant mechanical tension is identified as a biologically plausible contributor to pathological scar and adhesion formation following cesarean delivery. Subjective (e.g., POSAS, VSS) and objective (e.g., ultrasound elastography, transvaginal ultrasound) assessment tools are evaluated. Effective mechanotherapy interventions are unified into a proposed three-tier biomechanical framework: (1) reducing mechanical load transfer, (2) passive mechanical stabilization, and (3) mitigating external mechanical forces. However, a substantial evidence gap exists—most mechanotherapy studies focus on dermal scars, and direct validation for uterine niche rehabilitation is absent. Interventions such as silicone therapy, taping, and manual therapy are strongly applicable to superficial and adhesion-related components but remain hypothetical for myometrial defects.

**Conclusion:**

A biomechanical perspective is clinically relevant for post-cesarean scar care, particularly for abdominal wall scars and adhesion-related symptoms. The proposed three-tier framework offers a structured, phenotype-based approach to clinical reasoning. However, direct mechanotherapy for uterine niches remains unproven. This framework should be viewed as hypothesis-generating and a guide for superficial and adhesion-related management, not as a validated protocol for uterine isthmocele rehabilitation. Future prospective trials with ultrasound-based outcomes are urgently needed.

**Supplementary Information:**

The online version contains supplementary material available at https://doi.org/10.1186/s12905-026-04702-w.

## Introduction

Cesarean section remains one of the most frequently performed surgical procedures worldwide, with particularly high rates persisting across many Asian countries. In China, national data indicate an average cesarean rate exceeding 43.79% [1]. While neighboring regions show similar patterns. This epidemiological reality necessitates focused attention on both short-term and long-term postoperative complications.

Among these complications, postoperative scars—both abdominal wall scars and uterine scars—are common but often underrecognized.Abdominal wall scars result from fibroproliferative repair following incisions through the skin, subcutaneous fat, and anterior rectus sheath. Poor healing may manifest as hyperplasia, pruritus, pain, or, in rare cases, abdominal wall endometriosis. The uterine scar, located deep within the pelvis, represents the healing outcome of the incision in the myometrial layer of the lower uterine segment. Due to the region’s unique blood supply and myometrial structure, incomplete healing frequently occurs, leading to localized myometrial depressions known as “scar defects” or “niche shadows” [2, 3]. Cesarean Scar Syndrome (CSS) has emerged as a clinically significant yet often under-recognized condition affecting women’s long-term health. Characterized by chronic pelvic pain, dysmenorrhea, postmenstrual spotting, and secondary infertility, this condition represents a substantial burden on both patients and healthcare systems [4]. Beyond physical symptoms, the presence of prominent or symptomatic abdominal wall scars frequently leads to aesthetic concerns, psychological distress, and diminished quality of life—issues of particular cultural sensitivity in many Asian societies [5–7]. Scars are lasting signs of trauma, reflecting aspects of an individual’s identity and providing insight into the nature and timeline of the injury [8]. Importantly, these two scar types share a common etiology (surgical trauma) and are interconnected through the pathomechanism of adhesion formation.

Currently, clinical management of cesarean section scars often lacks standardization and may be approached reactively rather than through preventive or structured rehabilitative strategies. While the biological processes of wound healing are well-described, the translation of biomechanical principles into routine clinical practice remains limited. Clinicians frequently encounter patients with cesarean scars but may have limited guidance on systematic assessment or evidence-based intervention protocols.

This review addresses this clinical gap with three specific objectives: (1) To elucidate the biomechanical factors contributing to cesarean scars in a clinically accessible manner; (2) To evaluate assessment tools feasible in busy clinical settings; and (3) To propose a practical, stepwise rehabilitation framework that clinicians can implement directly. By bridging biomechanical science with clinical application, we aim to provide clinicians with concrete strategies to improve long-term outcomes for women undergoing cesarean delivery.Importantly, although CSS is fundamentally defined by a structural defect of the lower uterine segment (isthmocele), the biomechanical principles governing pathological wound healing—mechanotransduction, collagen remodeling, and mechanical load transfer—apply across all tissue layers from skin to myometrium. This review therefore adopts a biomechanical framework spanning superficial to deep tissues, while explicitly acknowledging that direct evidence for uterine niche rehabilitation remains limited. Wherever possible, we distinguish between evidence applicable to superficial scars and that which is hypothetically extended to uterine defects.

### Scope statement

The rehabilitation framework proposed in this review is primarily applicable to abdominal wall scars and adhesion-related manifestations of cesarean-related scars. It is not intended to directly guide mechanotherapy for the uterine niche (isthmocele) itself. Direct evidence supporting external mechanical interventions for myometrial defects remains absent, and such applications remain hypothetical unless explicitly stated otherwise.

## Methods

This review was conducted as a structured narrative synthesis integrating current knowledge on biomechanics and mechanotherapy into a clinical rehabilitation framework for cesarean-related scars. A formal systematic review with meta-analysis was not feasible due to substantial heterogeneity in study designs, populations, interventions, and outcome measures. Nevertheless, we adopted a systematic and transparent approach to literature search, screening, and synthesis to ensure methodological rigor. Reporting follows the PRISMA 2020 guidelines where applicable (see Additional file 1 for the completed checklist) .

### Search strategy

A comprehensive literature search was performed in PubMed, Web of Science, and Scopus from database inception to August, 2025, without date restrictions. Search terms combined three conceptual domains using Boolean operators: (1) cesarean-related scars (“Cesarean section,” “cesarean scar syndrome,” “isthmocele”); (2) biomechanics/mechanobiology (“scar biomechanics,” “mechanotransduction,” “adhesion”); and (3) assessment/intervention (“ultrasound elastography,” “silicone therapy,” “manual therapy,” “laser,” “acupuncture,” “taping,” “mechanotherapy”). The full search strategy for each database, including filters and limits, is provided as a Related file (Search Strategy). Additional publications were identified by manually screening reference lists of key articles and included studies.

### Eligibility criteria

Studies were included if they met the following criteria:

1. Study design: Basic science (in vitro/ex vivo/animal), observational (cohort, case-control, cross-sectional), interventional (RCTs, non-RCTs, case series with *n* ≥ 20), or systematic reviews.
2. Population: Scar healing, adhesion formation, or rehabilitation in cesarean section or comparable surgical incisions.
3. Interventions/exposure: Biomechanical aspects of scarring, assessment tools (subjective scales, elastography, MRE), or mechanotherapy (silicone, taping, manual therapy, laser, ultrasound, acupuncture, radiofrequency, tension-shielding devices).

#### Outcomes

Scar morphology, patient-reported outcomes (pain, itching, quality of life), biomechanical properties (stiffness, elasticity), or niche parameters (residual myometrial thickness, niche dimensions).

#### Language

English, peer-reviewed

#### Exclusion criteria

Studies focused solely on surgical techniques or obstetric outcomes without biomechanical/mechanotherapy relevance; sample size < 20 for clinical studies; conference abstracts, editorials, letters, and retracted publications; inaccessible full text.

### Study selection process

**Fig. 1:**
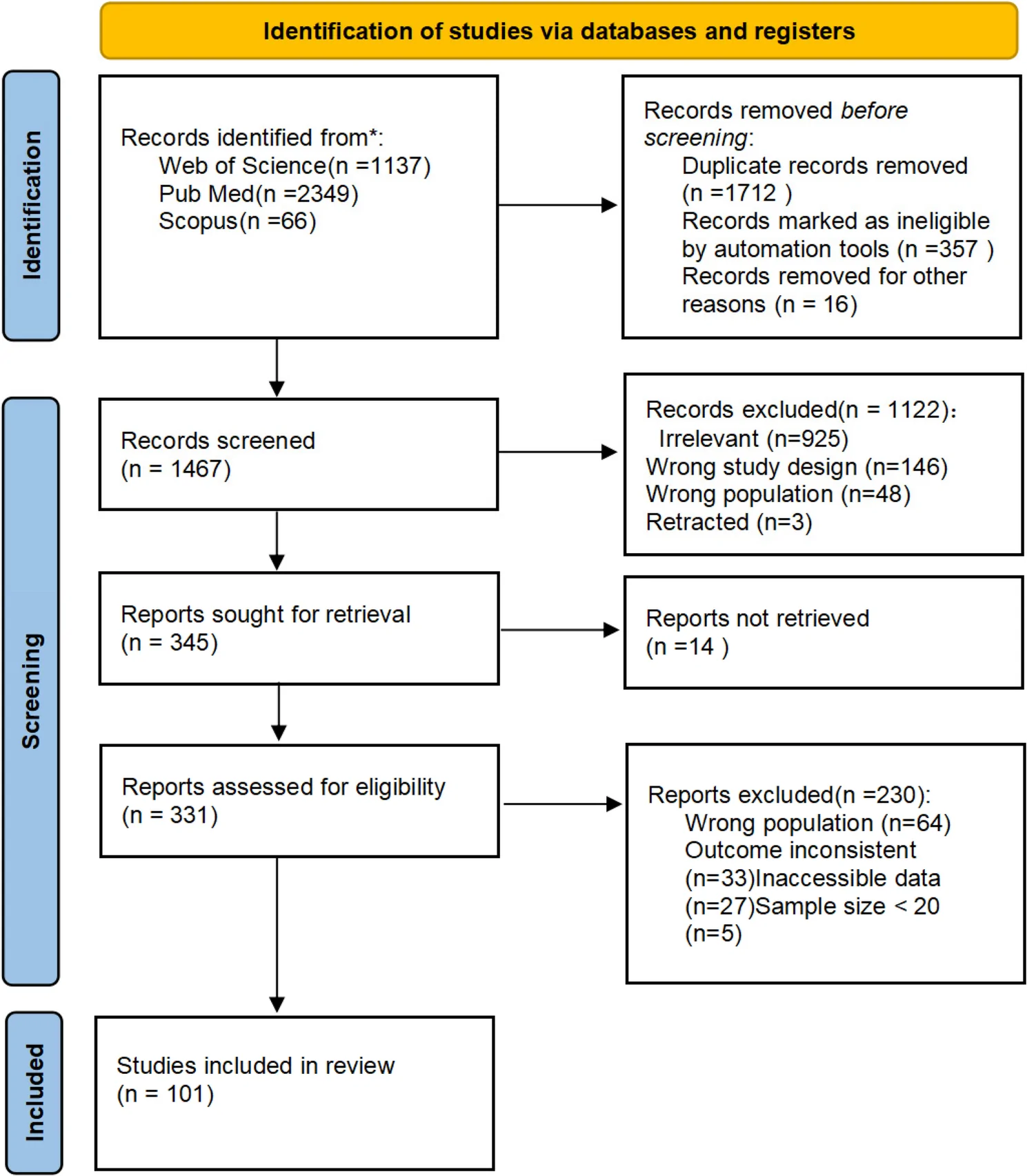
PRISMA 2020 flow diagram of the study selection process

The selection process followed the PRISMA 2020 flow diagram (Fig. 1) and was conducted in three stages:

- Stage 1 (Identification): All records were exported to reference management software; duplicates were removed using automation tools and manual verification.
- Stage 2 (Screening): Two reviewers (Z.Y. and H.J.Y.) independently screened titles and abstracts against eligibility criteria. Disagreements were resolved by discussion; if no consensus, a third reviewer (S.W.) made the final decision.
- Stage 3 (Eligibility): Full texts of potentially eligible reports were retrieved and independently assessed by the same two reviewers. Exclusion reasons were documented and reported in the PRISMA flow diagram.

### Data extraction

A standardized form was used to extract: bibliographic information (authors, year, journal); study characteristics (design, sample size, population, setting); intervention details (type, timing, frequency, parameters); outcome measures (scales, biomechanical parameters, imaging findings); key findings; and reported limitations. Two reviewers (Z.Y. and H.J.Y.) independently extracted and cross-verified all data.

### Evidence level classification and quality assessment

Given the heterogeneity of included study designs, no single quality assessment tool was applicable across all studies. We therefore adopted a dual approach:

#### Evidence level classification

Each study was assigned a level (I–IV or Mechanistic) based on design, adapted from the Oxford Centre for Evidence-Based Medicine hierarchy. These levels are summarized in Table 3.

#### Risk of bias

For interventional studies, risk of bias was assessed using the Cochrane Risk of Bias 2.0 tool (RCTs) or ROBINS-I (non-RCTs); observational studies were assessed using the Newcastle-Ottawa Scale. These assessments informed interpretation but did not serve as exclusion criteria. Publication bias is discussed in Sect.  6.3.

Detailed quality assessments are available from the corresponding author upon request.

### Data synthesis

**Fig. 2:**
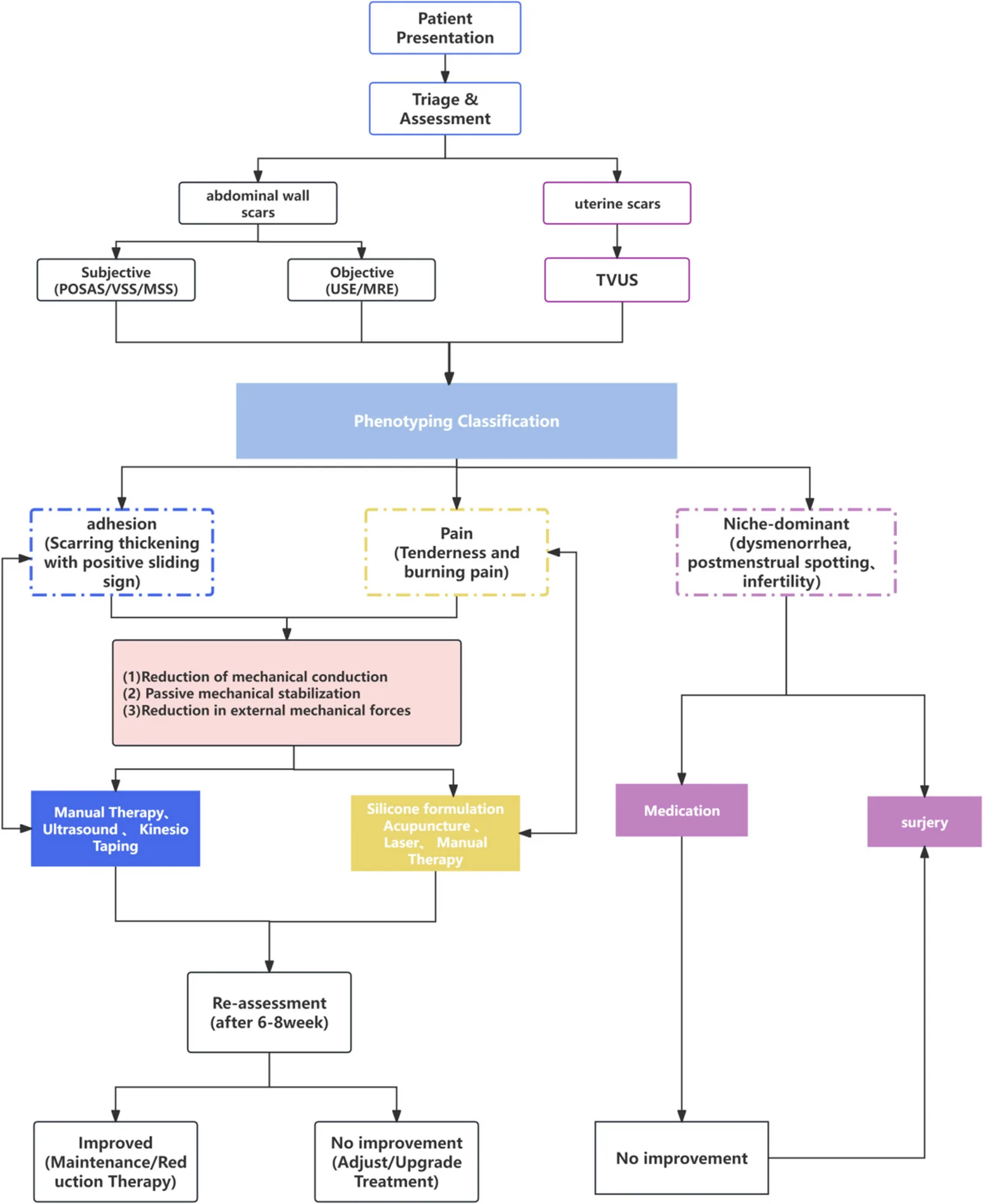
Proposed biomechanics-based clinical management pathway for cesarean-related scars

Quantitative meta-analysis was not feasible due to heterogeneity across studies. We therefore conducted a narrative synthesis organized around three thematic domains: (1) mechanical basis of scar formation; (2) assessment tools for scar biomechanics; and (3) mechanotherapy interventions. Findings were integrated into an original three-tier biomechanical rehabilitation framework (Fig. 2). To enhance clinical utility, we developed structured decision-support tools (Tables 1, 2 and 3).

**Table 1:** Clinical pathway for scar assessment: from subjective scales to objective technologies

	Assessment Type	Tool / Technology	Key Scar-Related Parameters	Clinical Application	Accessibility
1	Subjective (Patient-reported)	POSAS (Patient subscale)	Pain, itching, scar thickness, color, stiffness	Initial screening; captures patient’s perceived burden	+++
2	Subjective (Observer-rated)	POSAS (Observer subscale) / VSS / MSS	Pliability, vascularity, pigmentation, height; VSS focuses on pliability	Standardized clinical documentation; pre- vs. post-treatment comparison	+++
3	Physical examination	Palpation, skin glide test, stretch test	Reduced fascial glide; palpable induration; pain on stretch; scar width and hardness	Bedside assessment of adhesions and mechanical restriction	+++
4	Elasticity quantification (superficial)	Ultrasound elastography (USE)	Stiffness ratio (scar vs. normal tissue); predicts intra-abdominal adhesions	Objective baseline and monitoring; guides manual therapy intensity	++
5	Niche morphology (deep)	Transvaginal ultrasound (TVUS)	RMT (< 2.2–3.0 mm high risk); niche depth/width/shape; fluid accumulation	Diagnoses isthmocele; guides surgical vs. conservative management	+++
6	Complex/deep adhesion assessment	Magnetic resonance elastography (MRE)	3D stiffness map; fascial planes; utero-vesical adhesions	Reserved for complex cases with suspected deep adhesions	+
7	Predictive modeling (research)	Finite element analysis (FEA)	Stress/strain distribution; virtual testing of interventions	Surgical planning; personalized rehabilitation design	+

**Table 2:** Simplified clinical decision algorithm for cesarean-related scar rehabilitation

Step	Action	Key considerations
1.Assessment	History + physical examination + TVUS/USE	RMT, niche morphology, stiffness ratio, sliding sign
2. Phenotyping	Classify into one of three phenotypes	Adhesion-dominant / Niche-dominant / Neuropathic-dominant
3. Intervention selection	Match phenotype to mechanotherapy tier	Superficial → silicone/taping/manual therapy; Adhesion → manual therapy/tape; Niche → surgical referral if RMT < 3 mm
4.Reassessment	At 6–8 weeks	Monitor symptom change, scar mobility, stiffness reduction
5. Escalation or referral	If inadequate response or dominant niche	Consider surgical evaluation or alternative modalities

**Table 3:** Integrated decision support for mechanotherapy: evidence level, clinical parameters, and applicability to superficial vs. deep scars

Intervention	Evidence Level (Range)	Initiation Timing	Frequency / Duration	Primary Outcome	Applicable to Superficial?	Applicable to Niche?
Silicone gel/sheet	I (RCTs, meta-analyses)	After wound closure (day 7–14)	Daily (12–24 h), 3–6 months	Reduced thickness, pain, itching	Strong	Hypothetical
Paper tape	I (RCTs)	After suture removal (day 7–14)	Replace every 3–5 days, 3 months	Reduced height, pigmentation, itching	Strong	Hypothetical
Manual therapy	II-III (cohort, case series)	After epithelialization (week 3–4)	1–2×/week, 10–15 min, 6–8 weeks	Improved mobility, reduced stiffness	Strong	No evidence
Therapeutic ultrasound	II-III	Week 4–6	2–3×/week, 5–10 min, 8–12 sessions	Reduced stiffness, improved healing	Moderate	No evidence
Laser therapy	I-II	Week 2–4	1×/week, 4–6 sessions	Reduced pain, itching, pigmentation	Strong	No evidence
Acupuncture / Dry needling	II-III (systematic review)	Week 4–6	1–2×/week, 20 min, 6–10 sessions	Reduced inflammation, pain, itching	Moderate	No evidence
Radiofrequency	III-IV (case series)	Week 8–12 (mature scar)	Every 4–6 weeks, 3 sessions	Softening, reduced volume	Weak	No evidence
Tension-shielding device	I (RCT)	Immediate post-closure	Continuous for 8–12 weeks	Improved cosmetic outcome	Strong	Hypothetical
Intramuscular effect tape	II-III	Week 2–3	Continuous, replace 3–5 days, 4–6 weeks	Reduced tension, improved glide	Moderate	No evidence
Desensitization therapy	III-IV	Week 4–6	Daily home program, 4–8 weeks	Reduced allodynia, hyperalgesia	Weak	No evidence

Evidence levels are based on study design and do not necessarily reflect clinical efficacy specifically for cesarean-related scars, as most studies were conducted on general surgical or hypertrophic scars. Evidence level classification: For interventional studies, we classified evidence as Level I (RCTs/meta-analyses), Level II (non-randomized controlled trials/cohort studies), Level III (case-control studies), Level IV (case series), and Mechanistic (preclinical/in vitro studies)

A clear distinction was consistently maintained between interventions with direct evidence for superficial (abdominal wall) scars and those hypothetically extended to the uterine niche (isthmocele)—a distinction explicitly stated in the Scope Statement, applied in Results, and critically examined in Discussion.

### Ethical considerations

This review synthesizes existing published literature and does not involve new human or animal studies. Ethical approval was therefore not required.

### Study selection and characteristics

A systematic literature search identified 2,138 records across three databases (PubMed: *n* = 847; Web of Science: *n* = 690; Scopus: *n* = 590) and through hand-searching of reference lists (*n* = 11). Before screening, duplicates were removed (*n* = 723), records identified as ineligible by automation tools were excluded (*n* = 367), and records were removed for other reasons (*n* = 16), leaving 1,032 records for title and abstract screening.

Two reviewers (Z.Y. and H.J.Y.) independently screened titles and abstracts against the predefined eligibility criteria. Following this initial screening, 687 records were excluded for the following reasons: irrelevance to the topic (*n* = 72), wrong population (*n* = 265), and retracted publications (*n* = 5).

Of the remaining 345 full-text reports sought for retrieval, 14 could not be accessed. The remaining 331 full-text reports were assessed for eligibility by the same two reviewers independently, with disagreements resolved through discussion or consultation with a third reviewer (S.W.).

A total of 230 full-text reports were excluded, with reasons documented as follows: wrong population (*n* = 64), inconsistent outcomes (*n* = 33), inaccessible data (*n* = 27), sample size < 20 (*n* = 5), and wrong intervention (*n* = 101) .

Ultimately, 101 studies met the inclusion criteria and were included in this narrative synthesis. The detailed screening process is illustrated in Fig. 1 (PRISMA 2020 flow diagram).

## Mechanical factors in wound healing and scar formation

### Mechanotransduction as the unifying principle

Human skin is continuously exposed to internal and external mechanical forces. During wound healing, these forces regulate collagen synthesis and degradation by modulating matrix metalloproteinase (MMP) and tissue inhibitor of metalloproteinase (TIMP) expression [9]. Mechanical stimuli are converted into biochemical signals via a process termed mechanotransduction, mediated by receptors such as integrins and ion channels [10, 11]. Fibroblasts and myofibroblasts—the key effector cells in scarring—respond to these signals by secreting collagen and extracellular matrix (ECM) components, leading to progressive scar thickening and hardening [12–14]. Dysregulated mechanotransduction can disrupt normal healing, resulting in either excessive (hypertrophic scar, keloid) or insufficient repair [15].

Throughout the healing process, mechanical tension exerts phase-specific effects. Excessive tension during the inflammatory phase (first 48–72 h) disrupts balance, accelerating angiogenesis, nerve growth, and collagen overproduction [16]. Persistent mechanical stress during the remodeling phase (up to 1 year) impairs ECM remodeling and promotes hypertrophic scarring. Consequently, reducing mechanical forces in the wound environment improves healing and minimizes scarring—a principle that forms the foundation of mechanotherapy [17, 18].

### From cesarean incision to pathological scarring: a three-step logic

Scar formation signifies the conclusion of the final stage of wound healing [19]. From a practical clinical perspective, three sequential concepts are essential:

- Step 1 — Timing of healing phases. Wound healing proceeds through three overlapping phases: (a) the coagulation/inflammatory phase (0–72 h), (b) the proliferative phase (days 4–21), characterized by ECM deposition, angiogenesis, and re-epithelialization, and (c) the remodeling/maturation phase (week 3 to up to 1 year) [20]. Each phase has a distinct mechanical vulnerability profile.
- Step 2 — Mechanical vulnerability of the cesarean incision. The cesarean scar is located in a region of high mechanical stimulation—the suprapubic area of the lower abdomen. Mechanical stress arises from multiple sources: the orientation of the incision relative to skin tension lines (Langer’s lines), suture tension, and repetitive stretching during daily activities line [16, 21, 22]. These factors manifest clinically as widened, raised, or painful scars. The estimated incidence of postoperative scarring ranges from 40% to 70% [23–25]. Clinicians can assess this through simple observation and palpation during postpartum follow-up.
- Step 3 — From abnormal healing to CSS. When healing is disrupted, the clinical manifestations—chronic pelvic pain, dysmenorrhea, postmenstrual spotting, and infertility—are collectively termed Cesarean Scar Syndrome [26, 27]. A key clinical insight is that superficial scar abnormalities often correlate with intra-abdominal adhesions [28]. The adhesion rate increases with repeat cesarean deliveries: 32% after first, 42% after second, and 59% after three or more [29], with utero-bladder adhesions being the most common (41.8%). Palpable scar texture—particularly depressed, hypertrophic, or indurated scars—serves as an indicator of intra-abdominal adhesions [30–32]. A meta-analysis concluded that depressed scars are the most reliable predictor (sensitivity 38%, specificity 88%), outperforming flat or keloid scars [32]. Increased scar width and hardness also correlate with more severe adhesions [33]. These principles support a mechanism-informed clinical approach rather than purely symptomatic management.

### Intrauterine biomechanical modulation (limited evidence)

In addition to external factors, the intrauterine environment—such as hormonal status and uterine contractility—may also modulate niche behavior. However, clinicians should note that there is currently insufficient evidence directly linking intrauterine regulation to CSS outcomes; therefore, these factors should be regarded as hypothetical inferences rather than evidence-based research findings [34–36]. A personalized approach considering these intrauterine factors has been advocated within the PPPM (Predictive, Preventive, Personalized Medicine) framework [37].

### Organ–fascial–neuromuscular coupling: extrapelvic biomechanical effects of cesarean scarring

Beyond local scar mechanics, cesarean-related scarring can affect broader pelvic biomechanics through three interconnected pathways. First, uterovesical adhesions may restrict bladder and uterine mobility, leading to dysuria or pelvic pulling sensations. Second, visceral slide dysfunction between the uterus, bladder, and abdominal wall is assessable by dynamic ultrasound (sliding sign). Third, fascial tension transmission to the pelvic floor and lumbopelvic musculature may contribute to chronic low back pain or altered postural control. Conversely, persistent pelvic floor hypertonia or postural imbalance should prompt evaluation of abdominal scar biomechanics as a contributing factor [38–40].

## Scar characteristics and local symptoms

### Itching

Mechanical forces, including tensile loading and stress, significantly influence scar development and persistence. Itching, mediated through the TGFβ-interleukin (IL)-31 signaling axis, increases mechanical stress on the scar surface and stimulates vascular smooth muscle cell proliferation, leading to enhanced vascularity and collagen deposition [41].

### Pain

Chronic pain after cesarean section is multifactorial, involving both nociceptive and neuropathic mechanisms. A neuromechanical model integrates these factors through three interconnected pathways:

(1)The cesarean incision may directly injure or entrap the iliohypogastric, ilioinguinal, or genitofemoral nerves. Adhesions or scar contracture can create chronic nerve compression, leading to neuropathic pain [42]. (2)fascial-neural interactions whereby scar-induced stiffening alters afferent input and triggers central sensitization; and (3) central sensitization manifesting as hyperalgesia and allodynia, explaining widespread pelvic pain beyond the scar [43, 44].

### Infertility

By distorting pelvic anatomy and impairing fallopian tube mobility, adhesions are implicated in 20%–40% of female infertility cases and are the sole cause in approximately 15% [45, 46].

### Pelvic floor dysfunction

Adhesions can lead to urinary dysfunction [39], defecation disorders [47], and sexual dysfunction. The incidence of small bowel obstruction, while rare (0.1%–0.22%), increases with repeated surgeries [48].

### Impact on quality of life

The constellation of pain, physical restrictions, itching, and changes in body image significantly diminishes health-related quality of life and psychological well-being [16].

## Assessment of scar mechanics

A comprehensive assessment of scar biomechanics is essential for accurate diagnosis, prognosis, and treatment planning for Cesarean Scar. Assessment should follow a stepwise, multimodal approach, moving from subjective screening to objective quantification, and from superficial to deep tissue evaluation (Table 1). However, before engaging with specific assessment tools, it is crucial to recognize that the scar phenotype ultimately encountered is not a product of postoperative rehabilitation alone; it is fundamentally shaped by the initial surgical event.

Cesarean delivery represents a heterogeneous intervention. Factors such as emergency versus elective timing, the degree of tissue trauma and surgical handling, incision and closure techniques (including peritoneal layer management), bleeding or hematoma formation, and infectious or inflammatory complications can profoundly influence subsequent collagen remodeling, adhesion severity, stiffness, and pain characteristics. These surgical variables may also modulate individual healing responses and predict rehabilitation outcomes [22, 23]. Therefore, we propose that a focused surgical history—documenting these parameters—should serve as an initial predictive variable in the clinical reasoning pathway, helping to contextualize the biomechanical assessment that follows.

Following this pre-assessment step, subjective scales are then used to assess and monitor the progression of abdominal wall scars over time, as detailed below.

### Subjective assessment scales

Subjective scales are used to assess and monitor the progression of abdominal wall scars over time. These scales are typically free or low-cost, simple to administer, and easily utilized by healthcare providers. As a result, they are more frequently applied in clinical settings than objective scar measurement tools [45, 46].

Several clinical scar assessment scales are widely used, including the Vancouver Scar Scale (VSS), Visual Analog Scale, Manchester Scar Rating Scale, and the Patient and Observer Scar Assessment Scale (POSAS) [49]. Each tool has specific advantages and limitations; it is common practice to use multiple scales simultaneously to obtain a more comprehensive assessment.

The VSS, first introduced by Sullivan in 1990, assesses risk factors associated with hypertrophic scar development and evaluates treatment outcomes. It measures scar features such as scar vascularity, pigmentation, pliability, and height [46].

The POSAS, developed by Draaijers et al. in 2004, provides a structured method for assessing scar quality from both the clinician’s and the patient’s perspectives. POSAS evaluates parameters such as vascularity, pigmentation, firmness, pliability, surface area, and scar height. Unlike earlier scales, it also incorporates patient-reported symptoms such as pain and itching [50, 51].

Manchester Scar Scale (MSS): The MSS is used to assess the appearance and quality of scars resulting from surgery or trauma, providing a standardized tool for scar color, thickness, and flatness [46].

### Bedside physical examination

Beyond standardized scales, bedside physical examination provides immediate, low-cost information about scar mechanics. Key maneuvers include-Palpation: Assesses scar induration, tenderness, and texture [39].

### Objective imaging: from superficial to deep

#### Transvaginal ultrasound (TVUS) and ultrasound elastography (USE)

Transvaginal ultrasound (TVUS) is the primary imaging modality for assessing the uterine niche (isthmocele). Key parameters include residual myometrial thickness, niche dimensions (depth and width), niche shape, dynamic assessment, and bladder wall integrity. Additionally, TVUS is essential for preconception and early pregnancy assessment to exclude cesarean scar pregnancy and identify signs of incomplete uterine rupture, directly informing treatment intensity, prognosis, and safety counseling [52, 53].

Ultrasound elastography (USE) complements TVUS by mapping stiffness distribution along and beneath the scar. A higher stiffness ratio between scar and normal tissue predicts intra-abdominal adhesions, and USE can monitor treatment response [30, 54]. Integrating these findings enables three phenotype-based decisions: superficial/adhesion-dominant (RMT > 3 mm, normal niche, increased stiffness) → suitable for mechanotherapy; niche-dominant (RMT < 2–3 mm, deep niche) → limited role for external mechanotherapy, consider surgery; and mixed phenotype → individualized approach.

#### Functional ultrasound approaches

Ultrasound assessment offers more than morphological characterization of the niche; it provides functional biomechanical information that bridges imaging findings with clinical symptoms. Key functional parameters include dynamic assessment of visceral slide and organ mobility (e.g., the “sliding sign” between the uterus, bladder, and abdominal wall), evaluation of fascial layer interactions, and tissue stiffness mapping. These parameters, integrated with structural data from transvaginal ultrasound, enable a comprehensive biomechanical profile of the scar and its surrounding tissues.

This functional dimension is clinically relevant because Cesarean Scar Syndrome (CSS) is a heterogeneous condition in which identical symptoms—such as chronic pelvic pain—may arise from distinct pathomechanisms: a deep structural niche, tense intra-abdominal adhesions, nerve entrapment, or myofascial dysfunction. By correlating structural findings with functional mechanics, ultrasound serves as an integrative tool that enables phenotype-based differentiation and guides personalized intervention selection, moving beyond mere structural description toward mechanism-informed clinical decision-making [54, 55].

#### Magnetic resonance elastography (MRE)

MRE is a non-invasive technique combining MRI with propagating shear waves to generate three-dimensional maps of tissue stiffness. Its major advantage over USE is the ability to assess deep tissue structures, including fascial planes, myometrium at the scar niche, and adhesions between the uterus, bladder, and abdominal wall. This makes MRE particularly valuable for evaluating deeper pathomechanical disruptions contributing to pelvic pain and organ dysfunction. Due to high cost and limited accessibility, MRE is reserved for complex cases where deep adhesions are suspected [56].

### Ex vivo and laboratory techniques (mechanistic insight)

While not applicable to direct patient care, ex vivo techniques provide foundational knowledge on scar microstructure and micromechanics.

Atomic Force Microscopy (AFM): AFM operates at the nanoscale, using a sharp tip attached to a flexible cantilever to scan the tissue surface. By measuring the cantilever’s deflection as the tip indents the sample, AFM can calculate the local elastic modulus (stiffness) with extremely high spatial resolution [30, 57, 58]. This technique is crucial for understanding fundamental mechanobiology but is restricted to processed tissue samples [59].

### Computational modeling for prediction and planning

Finite Element Analysis (FEA): FEA is an engineering tool that simulates biomechanical systems by creating a digital model from medical images subdivided into small elements [60]. For cesarean-related scars, FEA can:

Predict risk of severe scarring or adhesions based on an individual’s biomechanical environment;

Identify stress concentrations around the scar that may drive pathological remodeling or cause pain; Guide personalized intervention by testing surgical closure techniques or rehabilitation strategies virtually [61, 62].

### Clinical integration: a multimodal pathway

The objective characterization of scar biomechanics is transitioning from a research tool to a clinical necessity. A multimodal, stepwise assessment pathway is recommended (Table 1):

- Step 1–2 (Subjective + physical examination): POSAS/VSS/MSS for patient-reported outcomes; palpation and glide testing for mechanical restriction.
- Step 3–4 (Routine imaging): USE for superficial stiffness quantification; TVUS for niche morphology and RMT.
- Step 5 (Complex cases): MRE reserved for suspected deep adhesions.
- Step 6 (Research/surgical planning): FEA for predictive modeling and virtual testing.

Integrating data from these technologies into the proposed rehabilitation framework enables a mechanistically informed, personalized management strategy for cesarean-related scars, moving from generic protocols to tailored interventions based on a patient’s unique biomechanical profile. Contemporary assessment methods are often highly sensitive, capable of detecting notable.

changes even before they are perceived by the patient [63].

## Mechanotherapy: biomechanical principles and clinical translation

Mechanotherapy refers to the application of controlled mechanical stimulation to promote tissue repair and remodeling. Injured and healthy tissues respond differently to mechanical loading. When the biomechanical properties of a healing wound are compromised—as occurs in cesarean section scars—targeted strategies are needed to restore the skin’s viscoelastic behavior. These strategies aim to improve mechanical load transmission and strain compatibility between scar tissue and surrounding skin, thereby enhancing both functional recovery and aesthetic outcomes [13, 57].

The principle that “function follows use” suggests that even minimal external force can induce functional realignment through mechanotransduction. Effective mechanotherapy therefore integrates mechanical loading with biochemical signals to optimize scar remodeling and promote tissue adaptation [13, 64]. In practice, mechanotherapy reduces mechanical stress at the wound site, improves load distribution, and enhances strain compatibility—contributing to better healing outcomes [19].

Following cesarean section, four primary types of physical stimuli affect the wound: tension (perpendicular stretch), compression (inward pressure), shear (parallel stretch), and osmosis (internal pressure maintaining cell expansion). Based on these biomechanical principles, scar treatment strategies can be grouped into three categories: (1) reduction of mechanical conduction, (2) passive mechanical stabilization, and (3) reduction of external mechanical forces [11, 13, 65, 66]. These three categories form the core of the proposed biomechanics-based clinical management pathway for cesarean-related scars (Fig. 2).

To facilitate clinical application of this pathway, a simplified decision algorithm is provided below (Table 2). This algorithm translates the biomechanical framework into a stepwise workflow following a three-step logic: Assessment → Phenotyping → Intervention selection, guiding the clinician from initial evaluation to targeted treatment and follow-up.For detailed criteria and stepwise guidance on when to escalate from conservative mechanotherapy to surgical evaluation, please refer to Sect.  From rehabilitation to surgery: a practical escalation pathway.

### Reduction of mechanical conduction

Early intervention during the inflammatory phase is essential for effective wound care in cesarean section. Scar prevention immediately after wound closure generally involves three key strategies: (1) relieving tension; (2) hydration, taping, or occlusion; and (3) the use of pressure garments [67] .Modulating mechanical forces throughout the healing process plays a significant role in preventing the progression to mature scar formation [68]. Reducing mechanical tension on healing wounds can help limit scar development.

#### Tension-shielding tool

The Embrace device is a tension-shielding tool made from a combination of silicone elastomers and medical-grade paper tape. It applies compressive force to the incision site to reduce tension and protect the area from mechanical stress. In a study by Gurtner et al., the use of a dynamic tension-shielding device on human abdominal incisions was shown to significantly improve the cosmetic outcome of the resulting scar [69, 70].

Hydrogel demonstrates stress relaxation and self-healing abilities, mimicking the natural characteristics of the extracellular matrix. Additionally, cross-linking with calcium ions induces spontaneous hydrogel contraction, facilitating wound closure and providing tension shielding around the wound site, thereby creating an optimal environment for scarless healing [71].

#### Ultrasound therapy

Low-intensity ultrasound, typically ranging from 0.125 to 3 W/cm^2^, provides non-destructive therapeutic effects and falls within the range commonly used in physiotherapy [72]. Through mechanical force, ultrasound generates a “hammering” action on tissues. It influences all three stages of wound healing: the inflammatory, proliferative, and remodeling phases [73]. The mechanical action of ultrasound helps regulate inflammation and minimizes excessive inflammatory damage. In skin tissue, low-frequency ultrasound has been shown to enhance the migration of dermal fibroblasts to the wound site, thereby facilitating healing following a 20-minute treatment [74].

#### High-intensity ultrasound

High-intensity ultrasound is generally defined as ultrasound with intensities exceeding 5 W/cm^2^and is associated with inducing coagulative necrosis, primarily used in high-intensity focused ultrasound ablation. When applied to scar tissue, this thermal ablation technique breaks down and softens the dense, disorganized collagen matrix, allowing for tissue resorption and reduced epidermal ischemia, which helps restore normal tissue thickness. Anastasova VN et al. found that focused ultrasound treatment leads to decreased tissue volume and scar density, realignment of collagen fibers, marked reductions in itching and pain, and improved pigmentation, including reductions in hyperpigmentation [72].

#### Laser therapy and Intense Pulsed Light (IPL)

The therapeutic effects of lasers and IPL rely on how light travels through tissue and how photon absorption leads to thermal, mechanical (photoacoustic), photochemical, and photobiological responses [75]. Lasers are considered a first-line treatment for traumatic scars and contractures. Optimal outcomes typically require a combination of different laser types, used either concurrently or alternately, depending on the clinical condition, treatment objectives, and specific treatment areas [76]. Strict sun protection is advised for at least 6 weeks before and after laser or IPL treatment. Therapy is usually initiated 1 week to 1 month following wound closure, with subsequent treatments spaced at intervals of at least 4 weeks or more [49, 77].

#### Radiofrequency therapy

Microplasma radiofrequency is a minimally ablative technique that utilizes radiofrequency energy to excite atmospheric nitrogen, generating a grid of high-energy plasma sparks. These sparks release heat as they return to a stable state, causing mild epidermal ablation and creating dermal microchannels within the scar tissue. The combination of ablation and thermal coagulation stimulates collagen regeneration and promotes both epidermal and dermal remodeling [78]. Immediate application of topical cooling following the procedure is essential due to localized heat accumulation in the treated area. Treatments are typically scheduled at 40-day intervals, with a complete course consisting of three sessions [79].

#### Acupuncture treatment

Acupuncture has emerged as a potential treatment modality for scarring, with several recent studies exploring its therapeutic effects. Acupuncture may facilitate wound healing by reducing localized inflammation, potentially minimizing scarring through regulation of ECM protein expression [80]. Increases in collagen, elastin, and epidermal thickness have also been reported in studies involving various scar types [81]. Interactions between cells and the ECM play a critical role in transmitting mechanical forces throughout tissues, and changes in collagen and elastin can alter the mechanical tension surrounding scars [13, 16].

The selection of acupuncture points for scar treatment varies among researchers. Typically, a 0.25 mm × 30 mm needle is placed around the entire scar or targeted to symptomatic areas if certain parts are “active” or painful. The two most sensitive spots are identified, with needles inserted 0.5–1.0 mm apart and positioned 0.5–1.0 cm from the scar at a 30–45° angle. Needle retention is usually around 20 min, adjusted based on patient sensitivity, and needle manipulation may be applied. Treatments are generally performed once or twice weekly until symptoms resolve or reach a plateau [81, 82].

### Passive mechanical stabilization

#### Tape

Passive mechanical stabilization of wounds over the long term has been employed to prevent wound expansion and subsequent mechanical transmission. Tape serves as a method to exert gentle pressure, which helps reduce local blood flow, promotes fibroblast differentiation, enhances scar tissue hydration, and limits collagen buildup. Clinical evidence consistently supports the use of low-tension tapes (such as Micropore™ or Steri-Strips™) to aid in closing linear surgical incisions by reducing skin tension. Recent studies indicate that stretchable tapes may positively influence hypertrophic scar development [83].

Tape is typically initiated at the time of wound closure, within the first week after surgery, or after suture removal. The tape is applied directly over the wound or scar and remains in place until it naturally detaches, generally every 3–5 days, at which point it is replaced [83].

#### Silicone-based therapy

Silicone-based therapy is considered the first-line treatment for managing proliferative scars, as it offers external mechanical support and helps reduce traumatic tension [84]. Akaishi et al. highlighted the role of silicone gel in minimizing tensile stress at scar sites and recommended its use as a tool for mechanical conditioning.This therapy alleviates tension within the scar by transferring stress from the junction between scarred and unscarred skin to the outer edge of the silicone sheet [85]. They are widely used due to their accessibility, affordability, ease of use, and minimal risk of adverse effects. Benefits include reduced scar thickness, pain, itching, and tenderness in severe proliferative scarring [86]. Silicone-based products can also be integrated with other scar management methods, including manual therapy, ultrasound, and laser treatments [87].

### Reduction in external mechanical forces

#### Manual therapy

Standardized soft tissue mobilization applies targeted pressure to scar tissue, accelerating collagen maturation and remodeling by disrupting fibrotic structures, thereby enhancing tissue flexibility and reorganizing the collagen matrix [88]. This intervention improves mobility, elasticity, and pressure-pain thresholds while reducing tissue stiffness in cesarean scars [89]. It also decreases vascular proliferation, hyperpigmentation, and scar height while improving surface texture and contour [90, 91]. Additional benefits include reduced tissue tension, increased post-treatment mechanical relaxation, improved interlayer mobility, and favorable changes in tissue viscoelasticity [92].

Massage should initially focus on areas surrounding the scar, with mobilization applied at each designated point on the cesarean scar for a total of 10 min (2 min per point at a frequency of 1 Hz). Clinically, light stretching motions adapted to the corresponding wound healing phase and incorporating intermittent oscillations (0.2 Hz) at the end of each amplitude are recommended [89]. Such manual therapy can begin once epidermalization is complete and the scar has sufficiently stabilized to support targeted manipulation [93].

#### Intramuscular effect patch therapy

Intramuscular Effect Tape is designed to replicate the thickness and elasticity of the skin, thereby assisting the muscular and lymphatic systems while offering mechanical support without limiting mobility. This tape generates small wrinkles or folds on the skin surface, lifting the skin slightly from the underlying tissue and thereby relieving pressure on the soft tissues beneath [94]. The gentle pressure exerted by the Intramuscular Effect Tape improves lymphatic circulation, activates dermal mechanoreceptors, enhances both sensory and mechanical input, promotes tissue nourishment, and decreases swelling [94]. When applied directly over wounds or scars, this tape reduces skin tension and helps prevent tissue overgrowth. Non-elastic tapes used to manage linear surgical scarring have been shown to significantly reduce scar height, pigmentation, and itching and are most effective when applied early in the healing process [95].

Clinically, manual therapy and taping should not be limited to the superficial scar but may need to address restricted fascial chains (e.g., scar–fascia–pelvic floor connections).Therefore, assessment of pelvic floor tension, lumbopelvic posture, and visceral mobility should guide the extent and focus of manual interventions.

The evidence levels, clinical parameters, and applicability to superficial versus deep scars for each of the above mechanotherapy interventions are summarized in Table 3.

## From rehabilitation to surgery: a practical escalation pathway

While the proposed framework emphasizes conservative mechanotherapy, a clear and practical pathway for escalating care is essential for clinical application. To guide decision-making, we propose the following structured stepwise approach, This pathway is designed to complement the conservative framework by providing clear, evidence-informed benchmarks for identifying patients who are unlikely to benefit from extended mechanotherapy and who may be candidates for surgical evaluation.

### Initial trial of conservative management

For patients with adhesion-dominant or mixed phenotypes, a structured mechanotherapy program (e.g., silicone therapy, taping, manual therapy) should be initiated for a defined period of 6 to 8 weeks. This timeframe allows sufficient opportunity for mechanobiological remodeling while avoiding prolonged ineffective treatment.

### Structured reassessment and indicators of treatment failure

At 6 to 8 weeks, patients should be re-evaluated using both subjective and objective tools. Treatment response should be considered inadequate if:

There is no significant improvement (≥ 30%) in patient-reported symptoms, OR There is no objective reduction in scar stiffness or improvement in tissue glide on physical examination or ultrasound elastography.

### Indicators for surgical referral

Immediate or expedited surgical consultation is recommended when any of the following criteria are met:

1. The patient presents with a niche-dominant phenotype with an RMT < 3 mm, particularly when associated with severe symptoms (e.g., chronic pelvic pain, postmenstrual spotting, or infertility);
2. Conservative management fails to achieve meaningful improvement after 8 weeks of structured therapy;
3. Imaging reveals a large, symptomatic isthmocele with fluid accumulation, or there is a history of cesarean scar pregnancy.

This structured escalation pathway ensures that patients unlikely to benefit from extended conservative care are identified early, thereby preventing unnecessary delays in receiving definitive surgical treatment. It also provides clinicians with clear, evidence-informed benchmarks for decision-making, enhancing the real-world applicability of the proposed framework.

## Discussion

### Summary of principal findings

This narrative review synthesized the pathomechanical basis of cesarean-related scarring and proposed a three-tier biomechanical rehabilitation framework (reducing mechanical conduction, passive mechanical stabilization, and mitigating external mechanical forces). The framework integrates subjective and objective assessment tools and provides a structured, mechanism-informed approach to scar management. However, several fundamental limitations must be acknowledged before this framework can be translated into routine clinical practice.

### The evidence gap: superficial versus deep scar management

A fundamental limitation of this review—and the broader literature—is the evidence gap between superficial scar management and uterine niche (isthmocele) rehabilitation [34, 53]. As stated in the scope statement, the proposed framework is primarily applicable to abdominal wall scars and adhesion-related CSS components, and is not intended to directly guide mechanotherapy for the myometrial defect itself.

Most mechanotherapy studies have focused on dermal or subcutaneous scars, with robust evidence supporting interventions such as silicone-based therapy [96]. However, extrapolating these findings to the uterine myometrial defect requires caution for three reasons.

First, tissue heterogeneity. The myometrium differs fundamentally from the dermis. Myometrial healing involves distinct cellular populations and mechanotransduction pathways [97]. Mechanical stretch activates specific signaling cascades in myometrial smooth muscle cells, including MAPK pathways, which differ from those observed in dermal fibroblasts. These differences suggest that interventions effective for dermal scars may not directly translate to the uterine niche.

Second, accessibility.Superficial scars are directly accessible to rehabilitation (e.g., manual therapy, taping, laser).The uterine niche, located deep within the pelvis, is not. Externally applied interventions likely have limited, indirect effects on the deep scar site, and no studies have directly validated external mechanotherapy for niche remodeling.

Third, outcome measurement.Superficial outcomes (POSAS, stiffness, height) are easily measured at the bedside.Uterine niche outcomes, by contrast, require transvaginal ultrasound (TVUS) to assess residual myometrial thickness (RMT), niche depth, and morphology [53, 98]-modalities rarely employed in rehabilitation studies [98, 99].

Therefore, consistent with our scope statement, readers should interpret the proposed framework as a guide for superficial and adhesion-related scar management. Direct application to uterine niche rehabilitation remains unproven and should be considered hypothesis-generating.

### Additional limitations of the evidence base

Beyond the superficial–deep evidence gap, several additional limitations affect the interpretability and generalizability of this review.

Lack of CSS-specific studies. Most interventions discussed were studied on general surgical scars, burn scars, or keloids, not specifically on cesarean scars or uterine niches. direct extrapolation may be invalid.

Small sample sizes and heterogeneity. Many studies, particularly those on manual therapy, acupuncture, and radiofrequency, are small (*n* < 50), uncontrolled, or lack blinding. Outcome measures vary widely (e.g., different scar assessment scales, different ultrasound parameters), preventing meta-analysis.

#### Conflicting evidence

For some interventions (e.g., therapeutic ultrasound), some studies report benefit while others find no effect. This may reflect differences in treatment parameters (intensity, frequency, duration) or patient selection. Currently, no consensus exists on optimal dosing.

#### Publication bias

Positive results are more likely to be published, potentially overestimating treatment effects. Negative or null studies on scar mechanotherapy are underrepresented in the literature.

#### Lack of long-term follow-up

Most studies report outcomes at 3–6 months. Long-term scar maturation (up to 1–2 years), recurrence rates, and durability of treatment effects are rarely reported.

#### No head-to-head comparisons

It is unknown whether one intervention is superior to another for a given scar phenotype, or whether combinations (e.g., silicone + manual therapy) are synergistic, additive, or redundant.

#### Framework validation status

The proposed three-tier framework is hypothesis-generating and derived from mechanobiological principles, not from validated clinical trials. Prospective studies are urgently needed to test its clinical utility across different scar phenotypes.

### Clinical implications and cautious interpretation

Despite these limitations, this review offers several clinically actionable insights. First, the strong evidence for superficial scar interventions (silicone, taping, manual therapy) supports their use for abdominal wall scars in patients with cesarean scars. Second, the framework provides a structured approach to clinical reasoning: assess phenotype (adhesion-dominant, niche-dominant, or neuropathic-dominant), match interventions accordingly, and reassess at 6–8 weeks. Third, for niche-dominant CSS with RMT < 3 mm and severe symptoms, surgical consultation should not be delayed by prolonged mechanotherapy trials.

However, clinicians should interpret recommendations for uterine niche rehabilitation as hypothesis-generating rather than evidence-based. External mechanotherapy for the uterine niche remains unproven; patients should be counseled accordingly.

### Future research directions

To bridge the evidence gap, future research should prioritize prospective trials focusing on cesarean scars with ultrasound-based outcomes (RMT, niche dimensions) as primary endpoints, alongside phenotype-stratified studies comparing mechanotherapy versus control across adhesion-dominant, niche-dominant, and neuropathic-dominant subgroups. Head-to-head comparisons of different mechanotherapy modalities for specific CSS phenotypes, dose-finding studies to establish optimal treatment parameters, and long-term follow-up (≥ 12 months) to assess scar maturation and symptom recurrence are also urgently needed. Furthermore, the proposed three-tier framework requires formal validation through multicenter prospective cohort studies or pilot randomized controlled trials. Beyond established mechanotherapy, future research should also explore image-guided minimally invasive techniques for selected scar-related disorders, such as ultrasound-guided hydrodissection or needle adhesiolysis for releasing fascial restrictions and nerve entrapments [55, 100]. Although evidence specific to cesarean-related scars remains preliminary, these approaches may represent a future direction within a personalized, phenotype-based management model, particularly for patients with localized adhesion-dominant symptoms who are poor candidates for or wish to avoid major surgery.

## Conclusion

In summary, this review provides a biomechanically informed, hypothesis-generating framework for post-cesarean scar rehabilitation. The proposed framework is grounded in established mechanobiological principles and supports a structured, phenotype-based approach to clinical reasoning. However, the evidence gap between superficial and deep scar management is substantial, and direct validation for uterine niche interventions is absent. As stated in our scope statement, this framework is primarily applicable to abdominal wall scars and adhesion-related complications following cesarean delivery. Clinicians should apply this framework cautiously, reserved for superficial and adhesion-related aspects of post-cesarean scars, and refer patients with dominant uterine niche pathology (e.g., residual myometrial thickness < 3 mm and severe symptoms) for surgical evaluation when indicated. Until prospective trials with ultrasound-based outcomes are available, the framework should be viewed as a guide for clinical reasoning and hypothesis generation, not as a validated treatment protocol for uterine isthmocele.

## Supplementary Information

Supplementary Material 1.

Supplementary Material 2.

Supplementary Material 3.

## Data Availability

No datasets were generated or analysed during the current study.
