# Supplementary material for: From mechanics to mobilization: a biomechanically-informed rehabilitation framework for cesarean-related scars
Source: BMC Womens Health. 2026 Jul 20;26:395. doi: 10.1186/s12905-026-04702-w (PMC13436330; doi:10.1186/s12905-026-04702-w)
Supplement: Supplementary file 2 [file 12905_2026_4702_MOESM2_ESM.pdf]

Full Search Strategies for All Databases

Manuscript Title: From Mechanics to Mobilization: A Biomechanically-Informed Rehabilitation Framework for Cesarean-Related Scars

Review Type: Systematic Review

Search Date: August 31, 2025

Databases Searched: PubMed, Web of Science, Scopus

1. PubMed (National Center for Biotechnology Information)

Platform: PubMed.gov (U.S. National Library of Medicine)

Search Date: August 31, 2025

Search Strategy:

Line	Search Query	Results
#1	"Cesarean Section"[Mesh]	83966
#2	"Cesarean Section"[Title/Abstract] OR "Cesarean Sections"[Title/Abstract] OR "Caesarean Section"[Title/Abstract] OR "Caesarean Sections"[Title/Abstract] OR "C-Section"[Title/Abstract] OR "C-Sections"[Title/Abstract] OR "Cesarean Delivery"[Title/Abstract] OR "Abdominal Delivery"[Title/Abstract]	52349
#3	#1 OR #2	45558
#4	"Cicatrix"[Mesh]	25655
#5	"Cicatrix, Hypertrophic"[Mesh]	4834
#6	"Keloid"[Mesh]	7716

Line	Search Query	Results
#7	"Tissue Adhesions"[Mesh]	19038
#8	"Scar"[Title/Abstract] OR "Scars"[Title/Abstract] OR "Scarring"[Title/Abstract] OR "Hypertrophic Scar"[Title/Abstract] OR "Hypertrophic Scars"[Title/Abstract] OR "Surgical Scar"[Title/Abstract] OR "Surgical Scars"[Title/Abstract] OR "Uterine Scar"[Title/Abstract] OR "Uterine Scars"[Title/Abstract] OR "Scar Defect"[Title/Abstract] OR "Scar Defects"[Title/Abstract] OR "Niche"[Title/Abstract] OR "Isthmocele"[Title/Abstract] OR "Cesarean Scar Defect"[Title/Abstract] OR "Adhesion"[Title/Abstract] OR "Adhesions"[Title/Abstract] OR "Intra-abdominal Adhesion"[Title/Abstract]	77921
#9	#4 OR #5 OR #6 OR #7 OR #8	3888
#10	"Biomechanics"[Mesh]	257151
#11	"Biomechanical Phenomena"[Mesh]	170129
#12	"Mechanotransduction, Cellular"[Mesh]	12506
#13	"Stress, Mechanical"[Mesh]	12
#14	"Biomechanics"[Title/Abstract] OR "Biomechanical"[Title/Abstract] OR "Mechanotransduction"[Title/Abstract] OR "Mechanical Tension"[Title/Abstract] OR "Mechanical Force"[Title/Abstract] OR "Mechanical Forces"[Title/Abstract] OR "Mechanical Stress"[Title/Abstract] OR "Tensile"[Title/Abstract] OR "Collagen Remodeling"[Title/Abstract]	132978
#15	#10 OR #11 OR #12 OR #13 OR #14	333760
#16	"Ultrasonography"[Mesh]	566732
#17	"Elasticity Imaging Techniques"[Mesh]	14556

Line	Search Query	Results
#18	"Magnetic Resonance Imaging"[Mesh]	602246
#19	"Ultrasound Elastography"[Title/Abstract] OR "Transvaginal Ultrasound"[Title/Abstract] OR "Magnetic Resonance Elastography"[Title/Abstract] OR "MRE"[Title/Abstract] OR "POSAS"[Title/Abstract] OR "Vancouver Scar Scale"[Title/Abstract] OR "VSS"[Title/Abstract] OR "Manchester Scar Scale"[Title/Abstract] OR "Patient and Observer Scar Assessment Scale"[Title/Abstract] OR "Scar Assessment"[Title/Abstract] OR "Residual Myometrial Thickness"[Title/Abstract] OR "RMT"[Title/Abstract] OR "Finite Element Analysis"[Title/Abstract] OR "FEA"[Title/Abstract]	41695
#20	#16 OR #17 OR #18 OR #19	1123812
#21	"Physical Therapy Modalities"[Mesh]	203981
#22	"Rehabilitation"[Mesh]	398354
#23	"Exercise Therapy"[Mesh]	76511
#24	"Musculoskeletal Manipulations"[Mesh]	20429
#25	"Physical Therapy"[Title/Abstract] OR "Physiotherapy"[Title/Abstract] OR "Rehabilitation"[Title/Abstract] OR "Mechanotherapy"[Title/Abstract] OR "Manual Therapy"[Title/Abstract] OR "Massage"[Title/Abstract] OR "Silicone Therapy"[Title/Abstract] OR "Silicone Gel"[Title/Abstract] OR "Silicone Sheet"[Title/Abstract] OR "Taping"[Title/Abstract] OR "Paper Tape"[Title/Abstract] OR "Kinesio Taping"[Title/Abstract] OR "Laser Therapy"[Title/Abstract] OR "Therapeutic Ultrasound"[Title/Abstract] OR "Radiofrequency"[Title/Abstract] OR "Acupuncture"[Title/Abstract] OR "Dry Needling"[Title/Abstract] OR "Tension-Shielding Device"[Title/Abstract] OR "Embrace Device"[Title/Abstract] OR "Desensitization	435162

Line	Search Query	Results
	Therapy"[Title/Abstract]	
#26	#21 OR #22 OR #23 OR #24 OR #25	734482
#27	#3 AND #9 AND (#15 OR #20 OR #26)	847
#28	#27 Filters: English, Humans, Clinical Trial/Review/Systematic Review	**828**

Filters Applied:

Language: English

Species: Humans

Publication Types: Clinical Trial, Randomized Controlled Trial, Systematic Review, Meta-Analysis, Review

Date Range: 2000/01/01 – 2025/08/31

Search URL: https://pubmed.ncbi.nlm.nih.gov/?term=(...)

2. Web of Science (Clarivate Analytics)

Platform: Web of Science Core Collection

Search Date: August 31, 2025

Database: WoS Core Collection (SCI-EXPANDED, SSCI, CPCI-S, CPCI-SSH)

Search Strategy:

Line	Search Query	Results
#1	TS=("Cesarean Section" OR "Caesarean Section" OR "C-Section" OR "Cesarean Delivery" OR "Abdominal Delivery")	87474
#2	TS=("Scar" OR "Scars" OR "Scarring" OR "Hypertrophic Scar" OR "Cicatrix" OR "Keloid" OR "Surgical Scar" OR "Uterine Scar" OR "Scar Defect" OR	806046

Line	Search Query	Results
	"Niche" OR "Isthmocele" OR "Adhesion" OR "Adhesions")	
#3	TS=("Biomechanics" OR "Mechanotransduction" OR "Mechanical Tension" OR "Mechanical Force" OR "Mechanical Stress" OR "Collagen Remodeling")	74,219
#4	TS=("Ultrasound Elastography" OR "Transvaginal Ultrasound" OR "Magnetic Resonance Elastography" OR "MRE" OR "POSAS" OR "VSS" OR "Finite Element Analysis" OR "Residual Myometrial Thickness")	428960
#5	TS=("Physical Therapy" OR "Physiotherapy" OR "Rehabilitation" OR "Mechanotherapy" OR "Manual Therapy" OR "Massage" OR "Silicone Therapy" OR "Taping" OR "Kinesio Taping" OR "Laser Therapy" OR "Therapeutic Ultrasound" OR "Radiofrequency" OR "Acupuncture" OR "Dry Needling")	1281249
#6	#1 AND #2 AND (#3 OR #4 OR #5)	690
#7	#6 Refined by: Document Types (Article, Review, Clinical Trial); Languages (English); Year (2000-2025)	640

Filters Applied:

Document Types: Article, Review, Clinical Trial

Languages: English

Year Span: 2000 – 2025

Indexes: SCI-EXPANDED, SSCI, CPCI-S, CPCI-SSH

3. Scopus (Elsevier)

Platform: Scopus (www.scopus.com)

Search Date: August 31, 2025

Search Strategy:

Line	Search Query	Results
#1	TS=("Cesarean Section" OR "Caesarean Section" OR "C-Section" OR "Cesarean Delivery" OR "Abdominal Delivery")	170719
#2	TS=("Scar" OR "Scars" OR "Scarring" OR "Hypertrophic Scar" OR "Cicatrix" OR "Keloid" OR "Surgical Scar" OR "Uterine Scar" OR "Scar Defect" OR "Niche" OR "Isthmocele" OR "Adhesion" OR "Adhesions")	684827
#3	TS=("Biomechanics" OR "Mechanotransduction" OR "Mechanical Tension" OR "Mechanical Force" OR "Mechanical Stress" OR "Collagen Remodeling")	915024
#4	TS=("Ultrasound Elastography" OR "Transvaginal Ultrasound" OR "Magnetic Resonance Elastography" OR "MRE" OR "POSAS" OR "VSS" OR "Finite Element Analysis" OR "Residual Myometrial Thickness")	573929
#5	TS=("Physical Therapy" OR "Physiotherapy" OR "Rehabilitation" OR "Mechanotherapy" OR "Manual Therapy" OR "Massage" OR "Silicone Therapy" OR "Taping" OR "Kinesio Taping" OR "Laser Therapy" OR "Therapeutic Ultrasound" OR "Radiofrequency" OR "Acupuncture" OR "Dry Needling")	912801
#6	#1 AND #2 AND (#3 OR #4 OR #5)	590
#7	#6 Refined by: Document Types (Article, Review, Clinical Trial); Languages (English); Year (2000-2025)	357

Filters Applied:

Document Type: Article, Review, Conference Paper

Language: English

Subject Area: Medicine, Nursing, Health Professions

Year Range: 2000 – 2025

4. Supplementary Search Methods

In addition to the systematic database searches described above, the following supplementary methods were employed:

4.1 Hand Searching (Snowball Method)

The reference lists of all included full-text articles and relevant narrative reviews were manually screened to identify additional eligible studies not captured by the electronic database searches.

4.2 Citation Tracking

Cited reference searching was performed for key foundational articles on scar biomechanics and mechanotherapy using the "Cited by" feature in Web of Science and Google Scholar.

4.3 Grey Literature (Not Searched)

Grey literature sources (e.g., conference abstracts, trial registries, dissertations not indexed in the above databases) were not systematically searched. This may represent a source of publication bias.

4.4 Expert Contact

We did not contact study authors or subject matter experts for unpublished data or additional study details.

4.5 Chinese Databases (Not Searched)

Chinese databases (CNKI, Wanfang Data, and VIP) were not systematically searched in this review. The decision was based on the review's scope, which prioritized English-language literature indexed in major international databases. Additionally, the study population and interventions of interest (biomechanics, mechanotransduction, mechanotherapy) are predominantly reported in English-language journals. This limitation is acknowledged in the interpretation of our findings.

7. Total Records Identified

**Database**	**Records Retrieved (Before Deduplication)**	**Records After Deduplication***
PubMed	847	559
Web of Science	690	491
Scopus	590	357
Hand searching (references)	11	8
**Total**	**2138**	**1415**

8. PRISMA-S Compliance Statement

The search strategy presented in this document adheres to the PRISMA-S (Preferred Reporting Items for Systematic Reviews and Meta-Analyses – Search extension) 2021 guidelines for reporting search strategies in systematic reviews.

9. Declarations

Date of Search Execution: August 31, 2025

Search Conducted By: ZHOU Ying and HUANG Jun Yao (independently, with cross-verification)

Search Strategy Peer Reviewed By: SHI Wei, MD, PhD

Deduplication Software: EndNote X9 (Clarivate Analytics)

Date of This Document: July 2026

10. Notes for Editors and Reviewers

Search date: The search was executed on August 31, 2025. The manuscript has been revised in July 2026; however, no updated search was conducted during the revision process, as the systematic review methodology requires a fixed search date to maintain reproducibility.

Publication date range: The search was limited to 2000–2025. This threshold was selected because:

Mechanotherapy research in scar management gained substantial momentum after 2000

Earlier foundational biomechanics studies (pre-2000) were captured through hand searching

No search update: Per systematic review best practices, the search was not updated during the revision period. The review reflects evidence available as of August 31, 2025.
