# Supplementary material for: From mechanics to mobilization: a biomechanically-informed rehabilitation framework for cesarean-related scars
Source: BMC Womens Health. 2026 Jul 20;26:395. doi: 10.1186/s12905-026-04702-w (PMC13436330; doi:10.1186/s12905-026-04702-w)
Supplement: Supplementary file 3 [file 12905_2026_4702_MOESM3_ESM.pdf]

PRISMA 2020 flow diagram for new systematic reviews which included searches of databases and registers only

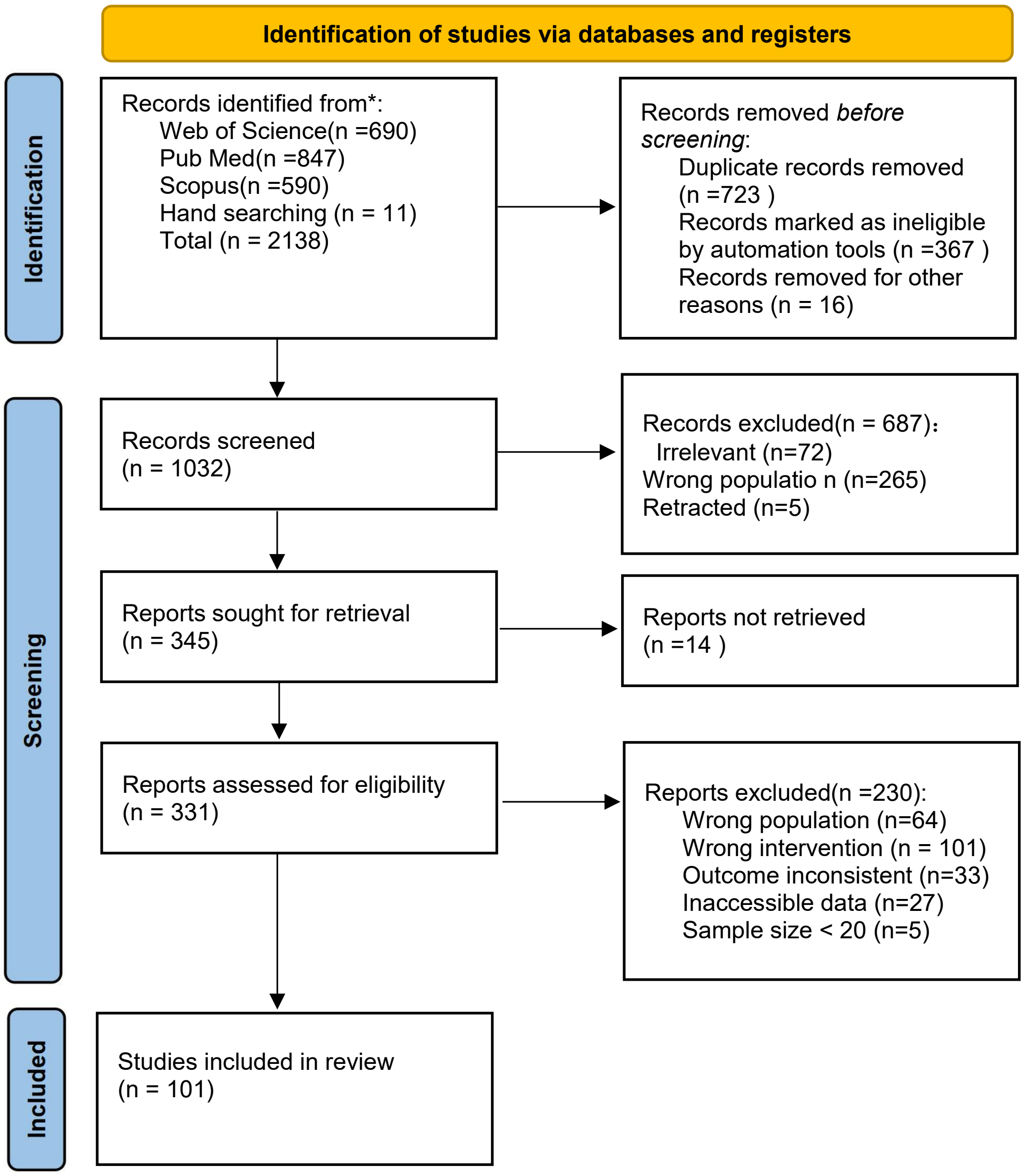

*Consider, if feasible to do so, reporting the number of records identified from each database or register searched (rather than the total number across all databases/registers).

**If automation tools were used, indicate how many records were excluded by a human and how many were excluded by automation tools.

Source: Page MJ, et al. BMJ 2021;372:n71. doi: 10.1136/bmj.n71.

This work is licensed under CC BY 4.0. To view a copy of this license, visit https://creativecommons.org/licenses/by/4.0/

PRISMA 2020 flow diagram for new systematic reviews which included searches of databases and registers only
